# Implementation of integrated services networks in Quebec and nursing practice transformation: convergence or divergence?

**DOI:** 10.1186/s12913-015-0720-8

**Published:** 2015-03-03

**Authors:** Caroline Longpré, Carl-Ardy Dubois

**Affiliations:** Centre for Training and Expertise in Nursing Administration Research (FERASI), University of Montreal, Montreal, Quebec Canada; Department of Nursing, Université du Québec en Outaouais, 5 Saint-Joseph Street, Room 3212, Saint-Jérôme, Québec Canada; Department of Nursing, University of Montreal, Montreal, Quebec Canada

**Keywords:** Processes, Integration of care and services, Care pathways, Professional practice, Nursing, Organizational and professional change

## Abstract

**Background:**

Even though nurses are expected to play a key role in implementing integrated services networks, up to now their practice in this regard has received very little research attention. The aim of this study is to describe the extent to which the evolution of nursing practice in Quebec in recent years has converged with the requirements and efforts involved in services integration.

**Methods:**

This descriptive study was carried out with 107 nurses working an integrated network of healthcare services in Quebec in four different care pathways: chronic obstructive pulmonary disease, autonomy support for the elderly, palliative oncology care, and mental health. Development model for integrated care (DMIC) was used, first, to examine the prevalence in each pathway of integrative activities, grouped into nine practice dimensions, and then to position each pathway in relation to the four phases of development for any integration process, as defined by the DMIC.

**Results:**

Only one pathway had reached Phase 3, which involves expansion and monitoring of integration, whereas the others were still in the preliminary Phases 1 and 2 characterized by initiative and experimentation. Only two dimensions out of nine (‘quality of care’ and ‘interprofessional teamwork’) were prevalent in all the pathways; two others (‘transparent entrepreneurship’ and ‘performance management’) were in none of the pathways, and the remaining five (‘patient–family centered care’, ‘result-focused learning’, ‘delivery system’, ‘commitment’, ‘roles and tasks’) were present to varying degrees.

**Conclusions:**

These results suggest that particular efforts should be made to bridge the significant gap between the pace of nursing practice transformation and the objectives of service integration. These efforts should focus, among other things, on the deployment of organizational, clinical, human, and material resources to support practice renewal and continuing education for nurses to prepare them for the requirements of integration.

**Electronic supplementary material:**

The online version of this article (doi:10.1186/s12913-015-0720-8) contains supplementary material, which is available to authorized users.

## Background

To deal with numerous challenges related, among other things, to population aging and the rising prevalence of chronic illnesses and mental health disorders, many jurisdictions are investing in strengthening the integration of health care and services. This is seen as a lever to ensure better accessibility and continuity of services and ultimately to improve public health [1-5]. In Quebec, starting in 2004, this integration took the form of a new organizational model in which the various resources in a given territory are linked together into a local health and social services network (LSN). At the heart of each LSN is a health and social services center (HSSC), created by merging local community health centers (CLSCs) with residential and long-term care centers (CHSLDs), and, in most cases, a hospital (CH). To meet its objectives, the HSSC is organized into service programs [6], which in turn are made up of different care and service trajectories or clinical pathways to address the needs of specific patient groups. The HSSC is complemented by a variety of service providers such as community organizations and pharmacies, youth protection centers, rehabilitation centers, family medicine groups (FMG), and municipal organizations. The LSN thereby created serves as the anchor point for developing local integrated services networks (ISN) that each provide a coordinated continuum of services for a defined population in the territory.

Despite the significant restructuring invested in setting up this model, recent reports have documented chronic shortcomings in terms of fragmentation, reduced access to services for vulnerable groups, and lack of service continuity [3,5,7-9]. The results of the 2011 Commonwealth Fund International Health Policy Survey showed that health services in Quebec fall short, to varying degrees, in accessibility, coordination, and continuity of care for patients with the greatest health needs. Quebec ranks last among Canadian provinces when it comes to users’ perceptions of the overall functioning of its healthcare system. In that survey, 16% of Quebec respondents did not have access to a family physician for primary care, as opposed to 9% in the rest of Canada; only 39% of hospitalized patients reported good coordination of care at discharge; and only 35% of respondents with a chronic illness reported that one person was responsible for all care related to their condition [9].

Some analysts have attributed these deficiencies to weaknesses in the integration model’s design and implementation [10-12]. Several studies have shown that an ISN only achieves its full potential when health professionals’ activities support the structural and administrative changes put forward by organizations [7,8,13]. However, in the recent reforms, the attention given to structural and administrative changes was often seen to be in stark contrast to the lack of investment in developing and renewing clinical and professional practices [5,8].

Given their responsibilities at all levels of care, nurses play a determining role in service coordination and care delivery [14]. They are a key link in the development of service programs and the achievement of underlying transformations. Implementing service programs involves, for example, developing referral mechanisms or new care protocols, introducing new roles to optimize nurses’ contribution, and developing renewed care activities that are more closely tailored to needs. It also includes developing collaborative practices that involve patients and families, nurses, as well as other service providers, levels of care, and organizations [15]. However, various studies have reported discrepancies between, on one hand, the imperatives of these integration processes and, on the other, nurses’ training. Some authors point out that integration calls for specific skills and competencies related to practicing in a network, in which nurses currently have little training [15-17].

While numerous studies have examined the integration process, nursing research in this area is still in its infancy, and to date there has been a relatively limited knowledge base to guide nursing practice innovations in this area, or to help in understanding the implications of these integration processes for nursing [7,15,18,19].

This study is intended to fill these gaps by looking at integration from the standpoint of nurses practicing in contexts undergoing transformation. Our objectives are: 1) to determine the extent to which nursing interventions in care pathway implementation converge with demands for greater integration of care and services; and 2) to determine the extent to which nursing practice is at similar or different phases of development in the integration process in different care pathways.

### Conceptual and theoretical bases

Several conceptual frameworks, each based on substantial literature, have been developed to support efforts to integrate care and services at different levels of the healthcare system. One well-known model is the Chronic Care Model (CCM) [20-23], which maps out chronic illness management activities in terms of six components: healthcare organization, service delivery systems, community resources, self-management support, decision support systems, and clinical information systems. In Canada, the Expanded Chronic Care Model [24], a CCM variant, emphasizes the community’s active role and assigns considerable importance to the determinants of health and to health promotion. The Innovative Care for Chronic Conditions framework developed by WHO [25-27] is another extension of the CCM that focuses on patients’ involvement in their own care, community resources, care organization, and the political environment. The Kaiser model, developed in California, prioritizes treatment according to a hierarchy of needs and patients’ risk level, with particular attention given to self-management support, interprofessional care management, and intensive case management for patients presenting complex conditions [28]. In Quebec, the integrated services network model [29] combines clinical (patient management procedures), governance (management, financial, and information systems), and values systems (beliefs, values, and interpretative schema that enable the actors to communicate and cooperate with each other). The pursuit of coherence among these systems results in integration of care, and in the normative, functional, and systemic integration of the clinical team.

These various models can be used to define the dimensions of service integration and understand how they relate to each other. Despite their specific contributions and recognized relevance, however, attempts to use these models for empirical examination of the evolution and implementation of integration projects are hampered by the scarcity of any validated instruments for this purpose. The only model for which any explicit effort has been made in this respect is the CCM, with the Assessment of Chronic Illness Care (ACIC), a tool for measuring certain healthcare processes, organizational structure, patients’ experience, and community involvement [30]. Several models presented here relate specifically to clienteles with chronic illnesses and are not necessarily designed to capture a variety of clienteles. Even though the underlying dimensions of these models may have to do with nursing practice, they do not represent the full scope of the nursing role [11].

**Figure 1:**
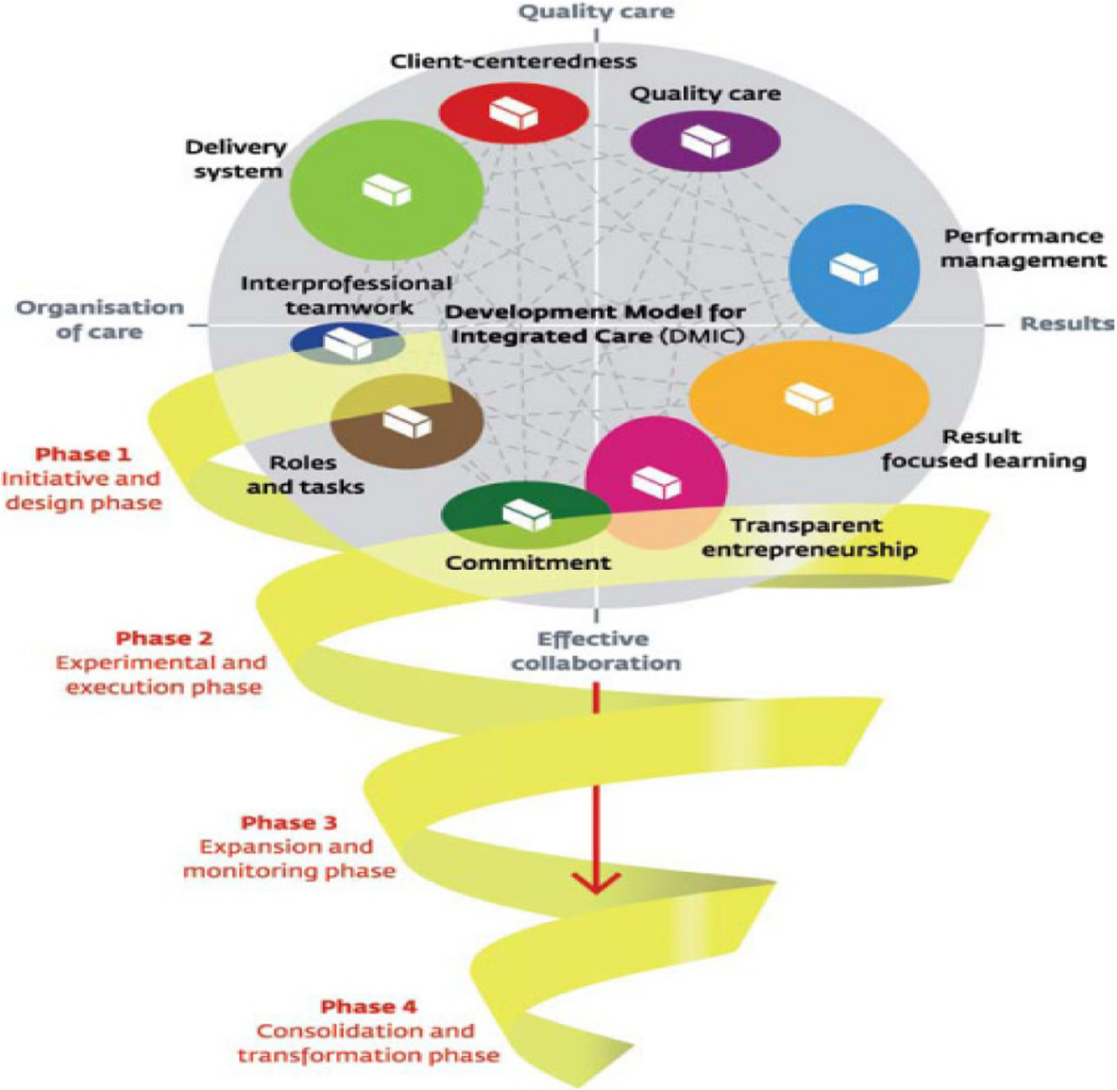
**Integrated care development model** **[** 19 **]** **.**

For this study, we selected as our reference framework the development model for integrated care (DMIC) developed by Minkman and colleagues, because of its conceptual contributions [31]. It is the only model that was specifically designed with a view to operationalizing nursing practices related to the integration process and that differentiates among the development phases of the process according to changes in practice (Figure 1). Unlike models centered on chronic illnesses, it can be applied to all clienteles and care pathways. The validity of the methodological process leading to its development has been demonstrated as has its theoretical validity [19,32,33]. Moreover, it has already been used, mainly in Europe, to evaluate and describe a variety of integration contexts, in such areas as traumatology, cardiology, and neurology services [19]. The present study provided an opportunity to test the model in the North American context and thereby to increase its external validity. In this model adapted to the Quebec context, nursing practice activities that contribute to the development of integrated care are operationalized as 89 integrative activities (*elements* in the DMIC model), organized into nine broad dimensions (*clusters* in the DMIC model): ‘client-family centered care’; ‘delivery system’; ‘performance management’; ‘quality of care’; ‘result-focused learning’; ‘interprofessional teamwork’; ‘roles and tasks’; ‘commitment’; and ‘transparent entrepreneurship’ (Table 1) [32]. The activities associated with these dimensions are ranked by complexity, making it possible to identify, using an analysis grid, four phases of development in the integration process: 1) initiative and design; 2) experimentation and execution; 3) expansion and monitoring; and 4) consolidation and transformation of the integration project [34]. The illustration of the model depicts the nine dimensions of integrative practice in interaction, following a narrowing spiral path from Phase 1 to Phase 4 as the complexity of integration mechanisms increases (Figure 1).

**Table 1:** **Dimensions of practice according to the DMIC**

**Dimensions**	**Definitions**
**Patient-family centered care**	Care delivery tailored and adapted to the needs of patients and families and information about care exchanged between patients/families* and care providers.**
**Delivery system**	Continuum of care organized for patients and families: coordination mechanisms and procedures to optimize all services; agreements and arrangements to provide care from admission to the end of the care episode.
**Performance management**	Measurement and analysis of services provided in a care pathway, based on established performance objectives, use of standard indicators, financial performance, accident and incident reports, and feedback; takes into account evaluations done by patients and families.
**Quality of care**	Evidence-based interdisciplinary care provided in response to patient-family needs and preferences.
**Result-focused learning**	Culture of continuous improvement of outcomes; involves definition of collaboration objectives, identification of potential improvements to care, learning strategies and knowledge exchange, and incentives to encourage improvement.
**Interprofessional teamwork**	Interdisciplinary work with a patient-family group defined by professionals who collaborate within organized and integrated teams.
**Roles and tasks**	Clarification of the roles and responsibilities of all partners involved in the services; effective collaboration among them and tasks well coordinated.
**Commitment**	Individual professionals’ commitment to defined objectives, intention to contribute, and knowledge regarding the nature of working within a care continuum.
**Transparent entrepreneurship**	Innovation, experimentation, leadership in matters of performance, financial agreements among partners, and partner transparency.

*Patient-family centered care: Expressed as ‘client-centered’ care in the DMIC, term adapted in the Quebec study to encompass patients and families.

**Care providers refer to persons rendering nursing, medical, and professional care to patients and families across the entire care pathway.

## Methods

### Design

For our descriptive study, we used a quantitative cross-sectional design [35] to identify nursing practices that were considered integrative and the dimensions underlying them in service program implementation, as well as to assess the extent to which the integration process had advanced in the different care pathways targeted. The study design was approved both by the research ethics committee of the University of Montreal (Project 11-085-CERSS-D) and by the ethics committee of the establishment being studied (Project 2213-11-06).

### Study environment

The selected environment was an HSSC in a semi-urban region that had been involved for several years in a major service restructuring to respond to the dramatic rise in numbers and significant aging of the population in its territory, in a context of limited professional resources. Because this HSSC had adopted a service program approach, which meant that interrelated services and activities were organized to respond to population health and social services needs or to the needs of groups of persons with a shared condition [6], and had developed integrated care pathways as a service organization model, it offered a relevant laboratory for the purposes of this study. This HSSC is representative of the recent reorganization of services in Quebec and encapsulates the challenges facing all HSSCs in this province.

### Service programs

Four of the HSSC’s five departments that were organized into service programs agreed to participate in the study. Each service program is subdivided into different pathways dealing with particular clinical situations and covers services provided by different establishments within the HSSC or the local health network (CH, CLSC, CHSLD, FMG). We selected four pathways: chronic obstructive pulmonary disease (COPD), autonomy support for the elderly (ASE), palliative oncology services (POS), and mental health services (MHS). Three main criteria guided our selection: 1) the existence of a critical mass of at least 30 respondents; 2) pathways situated at different levels of advancement (implementation date, specific issues); and 3) willingness and interest on the part of the management of the selected service programs to take part in the study.

### Nurse sample

The targeted population included all personnel with clinical functions except for patient care attendants (nursing assistant, technician, nurse clinician, counselor, navigator, liaison nurse, nurse practitioner) and management functions (coordinator, head nurse, assistant head nurse, director, manager). The criteria for inclusion were: being licensed to practice by their professional order, working in one of the selected pathways, and having worked full- or part-time, day or evening, for at least six months. We identified 200 nurses who met these criteria and invited them to join the study (COPD, n = 35; ASE, n = 70; POS, n = 40; MHS, n = 55).

### Study variables

Two main variables were measured. The first referred to nurses’ practices, identified as integrative activities that might advance care and service integration according to the DMIC. The second referred to level of integration, determined according to the four phases of the integration process. To construct a general profile of the respondents, we also collected data on sociodemographic variables, such as age, sex, role (clinical, management), education (post-secondary, university), location (CH, CLSC, CHSLD, palliative care, FMG, ambulatory services), and shift (day, evening, rotation).

### Data collection instrument

Data were collected using a questionnaire (see Additional file 1) made up of three distinct components. The first was an instrument to measure nursing activities, based on the DMIC [32]. It consisted of 89 items corresponding to activities considered integrative. For each item, nurses were asked to answer yes–no questions relating to, on one hand, the *relevance* of the activity to their practice, and on the other, its *presence*, that is, to what extent they considered the activity to be prevalent (or valued) within their service. The second component, used to determine the level of advancement of integration, was a validated grid developed by Minkman et al. [32] that positioned 40 activities (out of the 89 integrative activities) considered the most significantly representative of the four phases of the process (10 activities per phase) [34]. The third component consisted of 10 multiple choice and short answer questions added to the survey to capture information on sociodemographic variables.

The DMIC had to be translated from English into French. Adopting Vallerand’s [36] cross-cultural validation approach we translated the instrument and adapted it culturally for use in the Quebec context. We followed the standard methodological process of translation by two professional translators, consensus by a review committee, determination of conceptual equivalence by three expert groups, and a pre-test with nurses. KR-20 internal consistency analyses (Cronbach’s alpha used for dichotomous variables) showed significant consistency across the 89 activities (KR-20 relevance: 0.932, presence: 0.955), the nine dimensions (KR-20 relevance ranging from 0.9 to 1; presence ranging from 0.5 to 0.8), and the four phases of development (KR-20 ranging from 0.6 to 0.7).

### Data collection process

The researcher hand-delivered to each potential respondent identified (n = 200) a kit containing an information letter, the three-part questionnaire, an information pamphlet on ethical considerations, and a stamped return envelope. The information pamphlet explicitly stated that voluntary, anonymous return of the questionnaire constituted consent to participate in the research. To maximize response rate, reminders were provided two, three, and four weeks later, by telephone and directly within units.

### Data analysis

To address the first objective, we used descriptive statistics consisting of percentages and averages to examine concurrently the *relevance* and *presence* of the 89 activities. As set out in the model, an activity was considered relevant and/or present if at least 60% (≥60%) of respondents answered ‘yes’ to the corresponding statement. For an activity to be considered present, it must first have been considered relevant. A given dimension was considered prevalent if 60% or more of the activities associated with it were present. To address the second objective, descriptive statistics consisting of percentages and averages were used to determine the level of integration. According to the instrument for determining the development phase, a given phase has been reached when the score of activities considered significant for that phase is equal to or greater than seven out of 10 activities (score ≥70%) [34]. Descriptive and ANOVA variance analyses were conducted to produce the respondent profile and to examine associations between perceptions of activity presence and different variables, including respondents’ role, education, practice location, and work shift.

## Results

### Sample description

Of the 200 questionnaires distributed, 107 (n = 107; 54%) were returned by the respondents and used in this study. A high percentage of respondents were women with mainly clinical responsibilities, who worked days and were university trained. The practice locations represented were the CH, the CLSC, the CHSLD, FMGs, ambulatory centers (including outpatient clinics), and the palliative care center (Table 2).

**Table 2:** **Characteristics of the sample**

**Variables**	**Indicators**	**Staff (n)**	**%**
Population/pathway	ASE	35	32.7
	MHS	28	26.2
	POS	24	22.4
	COPD	20	18.7
	Total	107	100
Role/function	Clinical*	85	79.4
	Management**	22	20.6
Education	Post-secondary	34	31.8
	University - undergraduate	73	68.2
	University – master’s	8	7.5
	Total	107	100
Work shift	Day	88	82.2
	Evening	18	16.8
	Rotation	1	0.9
	Total	107	100
Practice location	CH	41	38.3
	CLSC	26	24.3
	CHSLD	20	18.7
	FMG	5	4.7
	Ambulatory care center***	13	12.1
	Palliative care center	2	1.9
	Total	107	100

*Nursing assistant, technician, nurse clinician, counselor, nurse navigator, liaison nurse, nurse practitioner.

**Manager, director, coordinator, head nurse, assistant head nurse.

***Refers to care and treatment provided in hospital for 12 hours or less, such as consultations, support services, or ambulatory care treatments (e.g. day medicine, day surgery).

### Prevalence of integrative activities

Of the 89 activities in the model, respondents assessed 87 to be relevant, across all pathways. The two activities assessed as non-relevant were ‘gathering financial performance data of the care chain’ (COPD) and ‘reaching agreements among care partners on discharge planning’ (POS). However, the percentage of prevalence of activities varied considerably from one pathway to another. Table 3 shows this percentage for each dimension by pathway.

**Table 3:** **Prevalence of integrative activities**

**Pathway % Activities present**	**ASE %**	**MHS %**	**POS %**	**COPD %**	**Total %**
**Dimension**					
Patient-family centered care	77.8*	66.7*	88.9*	44.4	66.7*
Delivery system	61.1*	44.4	88.9*	16.7	50.0
Performance management	37.5	12.5	43.8	18.8	25.0
Quality of care	60.0*	60.0*	60.0*	60.0*	60.0*
Result-based learning	75.0*	25.0	75.0*	33.3	41.7
Interprofessional teamwork	100*	66.7*	100*	100*	100*
Roles and tasks	62.5*	25.0	75.0*	37.5	50.0
Commitment	72.7*	9.1	72.7*	18.2	27.3
Transparent entrepreneurship	28.6	14.3	57.1	28.6	42.9
**Prevalent dimensions**	**N = 7/9**	**N = 3/9**	**N = 7/9**	**N = 2/9**	**N = 3/9**
**Prevalent activities**	**N = 54/89**	**N = 28/89**	**N = 64/89**	**N = 27/89**	**N = 40/89**

*Dimensions prevalent in the pathway.

As perceived by the nurses, more activities were present in the ASE and POS pathways, 54 and 64/89 respectively, than in MHS (28/89) and COPD (27/89). No pathway showed a prevalence of all nine dimensions. Two pathways (ASE and POS) showed seven prevalent dimensions out of nine, while MHS was limited to three and COPD to only two. ‘Quality of care’ and ‘interprofessional teamwork’ were the only two dimensions prevalent in all four pathways. The ‘patient-family centered care’ dimension was prevalent in three pathways (SAE, MHS, and POS); ‘delivery system’, ‘roles and tasks’, ‘commitment’, and ‘result-based learning’ were prevalent in two pathways (ASE and POS), while ‘performance management’ and ‘transparent entrepreneurship’ were absent from all four. Two-way ANOVA variance analyses—on pathways (inter-subject factor) and dimensions (intra-subject factor)—showed significant interaction between dimension and pathway factors (Table 4). In short, there is a significant percentage difference in “presence” between prevalent and non-prevalent dimensions, and the degree of difference depends on the care pathway.

**Table 4:** **Analysis of variance between dimension and pathway**

**Source**		**Degrees of freedom**	**Fisher**	**p-value**
Dimension pathway	Assumed sphericity	24	1.632	0.029
	Greenhouse-Geisser	17.935	1.632	0.048
	Huynh-Feldt	19.755	1.632	0.041

**Figure 2:**
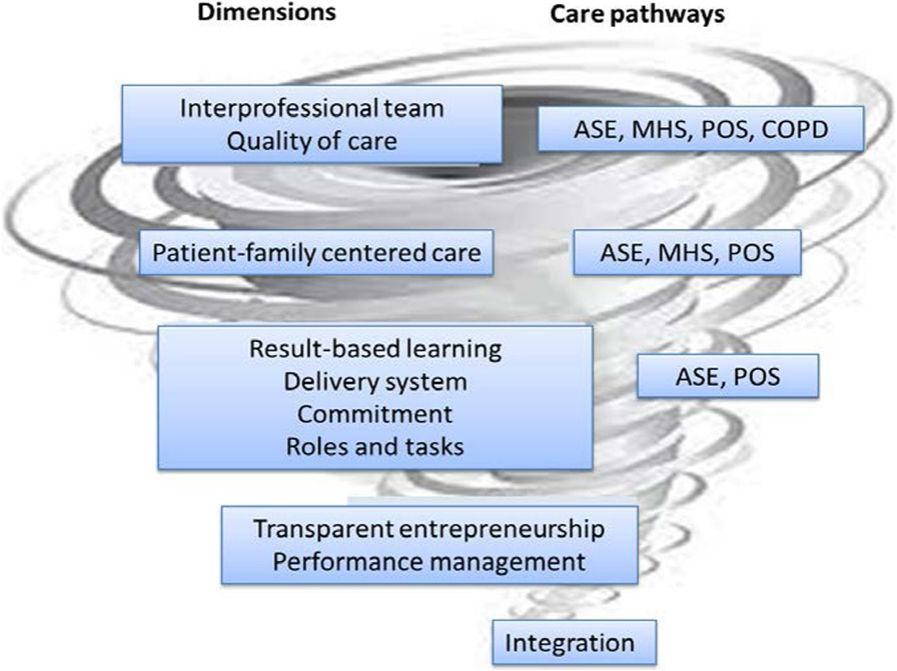
**Prevalent dimensions according to care pathway.**

Figure 2 presents the prevalent dimensions by care pathways, mapped out according to the DMIC model, depicting the widest part of the spiral as Phase 1 and the narrowest part as Phase 4 (Figure 1).

Figure 2 presents the prevalent dimensions as they correspond to care pathways, mapped onto the diagram illustration of Minkman’s [31] model presented in Figure 1 above. Dimensions that have only reached Phase 1 are situated at the widest segment of the spiral; they are present within all care pathways and represent the most accessible level of integration mechanisms. Conversely, at the narrowest extremity of the spiral are the dimensions assessed as non-prevalent in all pathways, which thus constitute the least accessible, or most complex, level of integration.

One-way analyses of variance were performed on sociodemographic data in relation to *presence* scores for each integrative activity. These analyses showed a statistically significant association for a threshold of 5% between *presence* scores, the role variable (p <0.001), and practice location (p <0.05). A non-significant association between *presence* scores and education and work shift variables was also detected. Furthermore, the analyses showed that nurses in management roles (manager, coordinator, director, assistant) identified on average a significantly higher percentage of *present* activities than did those with more clinical functions (nursing assistant, technician, clinical practitioner, counselor, nurse navigator) (p <0.001) (Table 5). Similarly, tertiary care nurses identified significantly more *present* activities than primary and secondary care nurses.

**Table 5:** **Significance of presence scores according to sociodemographic data**

**One-way ANOVA results**					**Levels comparisons**			
**Variables**	**N**	**%**	**Fisher**	**p-value**	**Levels**	**Diff.**	**Values**	**p-value**
Roles	106	99.0	17,937	<0.001	Manager (I)	I-J	0.195	<0.001*
					Nurses (J)			
Practice location	106	99.0	4,747	0.011	Primary (I) Secondary (J) Tertiary (K)	I-J	−0.021	1.000
						I-K	−0.156	0.011*
						J-K	−0.135	0.037*
Education	106	99.0	1.743	0.190	Post-secondary (I) University (J)			
Work shift	105	98.1	0,557	0.457	Day (J) Evening(E)			

*represents p <0.05.

### Level of advancement of integration process

**Figure 3:**
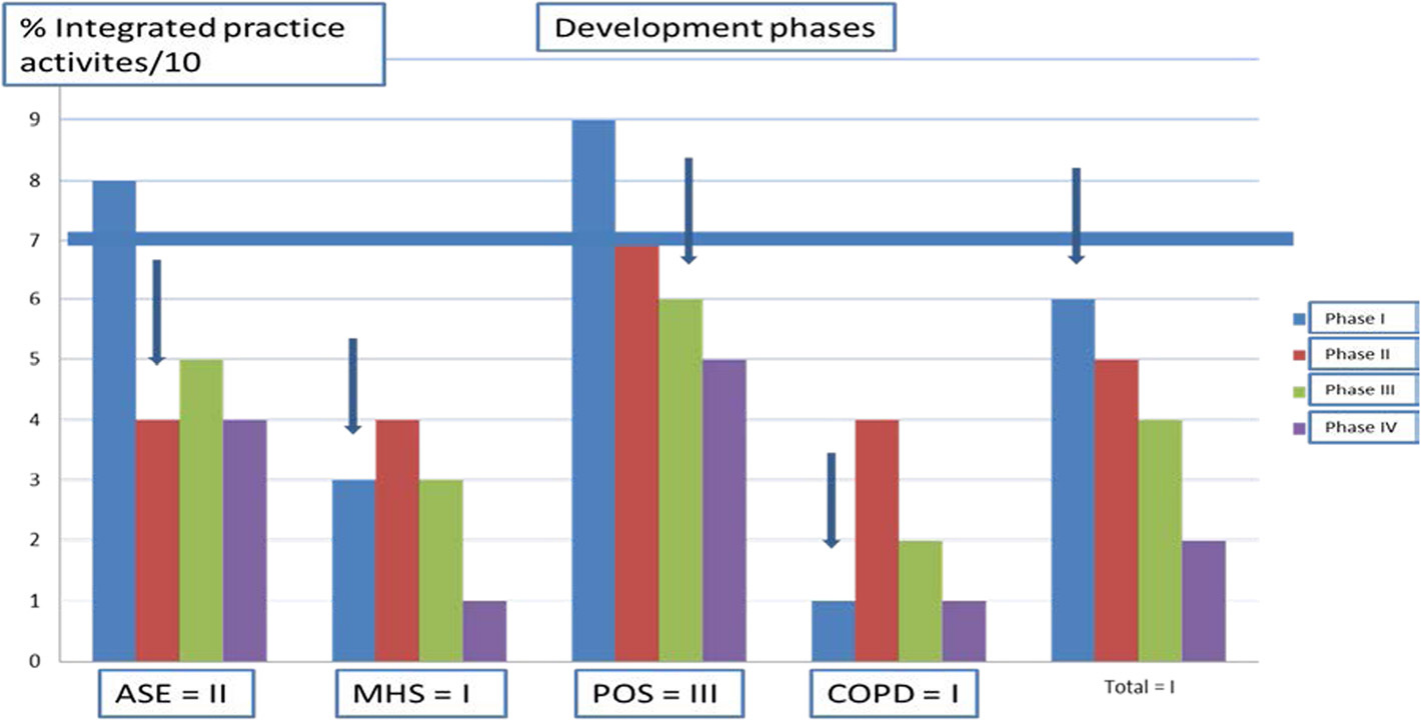
**Determining development phase.**

Figure 3 shows, for each care pathway, the number of activities present among the 10 activities characterizing each development phase (see Table 6 for detailed activities). MHS and COPD are seen to be still only in Phase I. With scores of 30% and 10% of activities present respectively, they do not meet the 70% threshold needed to progress to the next phase. ASE had completed Phase 1 (80% score), but remained in Phase 2 (40% score), and POS had reached Phase 3 (60% score), having completed Phases 1 (90% score) and 2 (70% score).

**Table 6:** **Integrative activities representing development phases**

	***ASE***	***MHS***	***POS***	***COPD***	**The integrative activities determining the phases of development**
**Phase 1**	X	X	X		2a: Reaching agreements on referrals and the transfer of clients through the care chain
	X	X	X		2d: Reaching agreements on procedures for the exchange of client information
	X		X		5b: Evaluating the services provided in collaboration with care partners
	X	X	X	X	6a: Defining with the care partners the patient-family group targeted by the care continuum
	X		X		8a: Defining the collaboration objectives in the care continuum
	X		X		8c: Ensuring leadership commitment from the care partners in the care continuum
	X		X		8d: Describing the roles and responsibilities of the leaders and coordinators in the care continuum
			X		8e: Formalizing the interdependency links between care partners and healthcare establishments
	X		X		9a: Committing together to achieving the clinical objectives targeted by the care continuum
					8b: Signing collaboration agreements between care partners
**Phase 2**			X		2 h: Using common care and treatments plans across the entire care continuum
		X	X	X	2p: Using one or more specialized nurses to provide services in the care continuum
	X	X	X	X	4d: Respecting evidence-based practice standards
	X	X	X	X	6b: Working in interdisciplinary teams
	X		X		7b: Making adjustments as needed to the roles of the various care partners
	X		X		7c: Ensuring care partners know each others’roles and responsibilities
					7f: Encouraging partner meetings on the whole care continuum
					3 g: Following up on results obtained while developing the care continuum
					2 m: Agreeing on leave plans among care partners
					3d: Gathering information on continuum logistics (e.g. patient traffic, wait times, delays) within the continuum
**Phase 3**	X	X	X		1c: Determining the client-family’s required care plan (ITP and IIP) with the care partners
	X	X	X	X	1f: Adjusting services throughout the care continuum to respond to specific patient-family needs
	X		X		2e: Accessing the databases of all care partners in the care continuum
	X		X		3i: Ensuring follow-up of all accident/incident reports related to the care continuum
	X	X	X	X	5j: Accessing training programs and learning opportunities for care partners
			X		5 l: Promoting exchanges among care partners to make innovations in services provided in the care continuum
					3j: Applying a systematic method to evaluate approaches used (e.g. care delivery) and results obtained
					7 g: Agreeing on how to introduce and incorporate new care partners into the care continuum
					4e: Ensuring that client representatives participate in care continuum performance evaluations
					3 m: Demonstrating to care partners the effect of the continuum on the care provided
**Phase 4**	X		X		3i: Ensuring follow-up of all accident/incident reports related to the care continuum
	X		X		5e: Sharing knowledge among care partners on effective organization of services in the care continuum
			X		5 h: Offering incentives to care partners to encourage them to achieve quality objectives
	X	X	X	X	5j: Accessing training programs and learning opportunities for care partners
	X		X		8 h: Reaching agreements on each care partner’s specific areas of care (who does what)
					9 g: Having a single block of funding to distribute across the continuum of care
					5 k: Sharing with care partners the results of achieving continuum objectives
					8 k: Meeting external stakeholders: government agencies, community organizations, etc.
					1i: Using standardized care protocols (e.g. systematic follow-up) adapted to client groups with specific needs
					9c: Agreeing on setting up a financial budget for the care continuum

## Discussion

The results of this study—whose objectives were to determine the extent to which nursing interventions converge with the requirements of greater integration of care and services and the extent to which nursing practices reflect convergent or divergent phases of development of the integration process in different care pathways—reveal that the efforts invested over recent years in Quebec’s health network have been slow to manifest at the level of nursing practices. Although nurses confirmed that 98% of the integrative activities proposed by Minkman et al. [32] are relevant to their practice, only 40% of these activities were assessed as being actually present in their practice environments. Despite the low percentage of present activities, certain dimensions that would denote the integration of care were more prevalent than others within the pathways. ‘Interprofessional teamwork’ and ‘quality of care’ were prevalent and convergent in the four pathways examined, and ‘patient-family centered care’ was prevalent in three pathways. These three dimensions’ stronger prevalence could be due to the increased emphasis placed in recent years on the concepts of collaboration and partnership in intra- and interprofessional teams [37], as well as on the concepts of quality of care and patient-centered care [38], both in nurses’ initial and continuing education and in the vision of nursing promoted in the organization.

With regard to the ‘interprofessional teamwork’ dimension, all stakeholders have come to recognize that interdisciplinarity is essential to improve accessibility and quality of care and services, clinical outcomes, financial results, staff retention, user satisfaction, and patient safety [39,40]. In fact, numerous specific initiatives have been launched within organizations to strengthen this dimension. One example is the large-scale, mandatory implementation of the Therapeutic Nursing Plan (TNP) and the Interdisciplinary Intervention Plan (IIP), which provide tools to facilitate collaborative relationships, exchange, and consultation among healthcare professionals [41]. Such mechanisms for interdisciplinary collaboration are instrumental in supporting integrated care, as they enable cooperation and sharing of vision not only between nurses, but also between nurses and other professionals or managers.

The ‘quality of care’ dimension has also received increased attention in recent years, as reflected in a number of quality assurances mechanisms, including the user complaint review system, the ombudsman role, risk management programs, accreditation systems, and coroner investigations into the circumstances of certain deaths. Ministerial directives, on the other hand, govern the application of certain interventions, such as the use of restraints, isolation, or chemical measures [42].

The ‘patient-family centered care’ dimension was prevalent in three pathways. The complexity of certain health situations calls for the mobilization of care and services from a variety of partners as well as from patients and families themselves. The trend now is to offer integrated care and services in partnership with the patient and family, so they can take an active role, inasmuch as they are able, in decisions affecting them. The prevalence of this dimension in three pathways can be explained by a growing, although undoubtedly still insufficient, integration of this approach into clinical settings based on partnership with patients and their families. A survey of Quebec initiatives in this area shows an increasingly active participation of ‘partner’ patients and their families in clinical team meetings, training in illness management and treatment, development of care plans and IIPs, and even in the training of health professionals [43].

The ‘commitment’, ‘roles and tasks’, ‘delivery system’, and ‘result-focused learning’ dimensions, all of which are also important to a care integration process, were not assessed as prevalent in the MHS and COPD pathways, in contrast to the ASE and POS pathways. Considering these results, we could hypothesize that, given the nature of these dimensions, actualizing them might require that the integration project had achieved a certain maturity and level of advancement. The pathways for which these dimensions were not prevalent were positioned at less advanced phases of development. In these two pathways, commitment, role clarification, result-based learning, and implementation of a care delivery system may have been hampered by inadequate knowledge of the changing nature of team work, delays in setting up the tools and structures needed to meet integration objectives, recurrent problems with information systems and accountability mechanisms, or even just the time needed for actors in the health network to adjust to these professional and organizational changes.

The ‘transparent entrepreneurship’ and ‘performance management’ dimensions, on the other hand, converged. They were not prevalent in any of the care pathways studied. We concluded that these dimensions had more to do with notions of functional integration, which, according to Contandriopoulos et al. [29], combines financial, information, and network management systems comprehensively to create the shared governance required to coordinate integrated clinical teams’ practices and operations. Even though the nurses generally recognized the relevance of activities associated with these dimensions, they did not identify them as being present in their settings. It could be that the integration process had not reached the level of advancement needed for effective implementation of activities related to these dimensions, or perhaps that not enough resources had been invested to support such activities. Presumably the nurses knew less about, and therefore engaged less in, practices corresponding to these dimensions because these activities were further removed from clinical practice or were more complex. In this respect, it should be noted that nurses in this study who had management roles indicated the presence a significantly higher number of integrative activities than did those in clinical roles. Many clinical nurse respondents said they were not familiar with the concepts and notions underlying integration, which they saw as belonging more in the management arena. They indicated they were not very aware of the principles of integration. None of the nursing assistants invited to participate in the study completed the questionnaire, as they said they did not feel they were involved in the subject of integration. The ‘theory of bureaucratic caring’ [44] is an attempt to bring management and clinical functions closer together; as such, the contemporary view of practice stresses the importance of partnership and of consistency in the views and language of care providers, service managers, and administrators.

Taken together, these results reaffirm the conclusions reached in the literature to the effect that the practice changes required for service integration occur slowly and sometimes even with difficulty [5,11,45]. Some authors have highlighted the difficulty of implementing case management systems or population-based chronic illness management systems, as compared with implementing specific clinical interventions such as clinical practice guidelines [18,46], or the challenges of implementing initiatives to harmonize practices, which require time, adaptation, and open-mindedness [47]. Other studies have shown the problems involved in coordinating work between organizations [47], or in implementing new interventions that are in sharp contrast to the silos in which organizations and professionals currently operate. One study on nursing resource utilization models and scope of nursing practice suggested that nursing resources are generally under-utilized in organizations and that certain key dimensions of the nursing profession are frequently under-utilized, such as communication and coordination, quality of care, and the updating and application of knowledge [48]. This would appear to be due, in part, to the fact that nurses are not always prepared, professionally or personally, to contend with the challenges presented by changes in service organization [15,49-52].

Despite healthcare organizations’ investments over recent years in efforts to increase integration, this study’s findings showed that, from a nursing perspective, the care pathways were generally still only in the preliminary phases of developing integration processes. The MHS and COPD pathways were at the least advanced level of integration, i.e., Phase I (initiative and design). These pathways had gotten off to a slower start and were still in the process of conceptualizing service organization and developing collaborative agreements with other settings. In the MHS pathway, for example, few interprofessional collaboration mechanisms had been developed in the hospital’s psychiatry unit, as opposed to the very active collaboration mechanisms already operating at the primary and secondary service levels. For the COPD pathway, which was at the very beginning of its development, no agreements had yet been reached on structural, organizational, and professional procedures for integration among the settings encompassed by the HSSC, such as the COPD clinic, the inpatient medical unit, the FMGs, and the CLSC. There was still no clear understanding regarding how the pathway was to be organized and what each partner would contribute. Also, the Ministry guidelines were not as directive, nor as clearly laid out, as they were, for example, for the POS pathway. The ASE pathway, although in Phase 2 (experimentation and execution), was also relatively little advanced. Procedures for collaboration and coordination were still unclear. The slow progress in getting this pathway started may have been due to long-standing and deeply entrenched practices, as well as to certain stakeholders’ resistance to change.

The POS pathway was the only one whose integration was relatively more advanced, i.e., at Phase 3 (expansion and monitoring). It showed continuous development and a certain maturity of the care continuum, especially with regard to innovation and the pursuit of improved outcomes [34]. This level of advancement can be explained by key developments that included the implementation of a provincial cancer program [53] and the introduction of nurse navigators and a nurse assigned to palliative care. These new roles were dedicated to integrative activities, such as: participating actively in the interdisciplinary approach and collaborating on the development and implementation of IIPs; ensuring continuity of care; serving as a resource for clients, families, and practitioners; directing patients and staff toward other professionals on the interdisciplinary team, as required; and guiding patients through the network [54]. There is widespread agreement in the literature that these recent roles, and others being developed, are having a very significant impact on care integration.

Another conclusion drawn from the results of this study was that the integrative activities were divergent in terms of their pace of progress (Table 7). Activities corresponding to the ‘quality of care’ and ‘patient-family centered services’ dimensions were prevalent for the pathways that had achieved at least Phase I in their development (except for ‘patient-family centered services’, which was not prevalent for COPD). It is reasonable to assume that these dimensions would begin to take shape more quickly or efficiently than the others at the start of any integration project and, as such, would lay the foundations for such projects. This might be because nurses have more knowledge and skills related to these dimensions, or because there are better integrated organizational procedures in place that encourage their prevalence.

**Table 7:** **Development phases and prevalent dimensions**

Phase 1	Quality of care
	Interprofessional teamwork
	Patient-family centered services
Phase 2	Result-based learning
	Delivery system
	Roles and tasks
	Commitment
Absent dimensions	Performance management
	Transparent entrepreneurship

The ‘roles and tasks’, ‘commitment’, ‘delivery system’ and ‘result-based learning’ dimensions were prevalent for the care pathways that had reached at least Phase 2 in their development, i.e., POS and ASE.

These findings indicate the scope of investment required to ensure nursing practice is aligned with the requirements of service integration. Investments in basic and continuing education would raise awareness and help prepare nurses and other stakeholders, regardless of status or function, to cope with the requirements associated with developing the integration process in relation to emerging professional practices. Such training should cover a variety of topics of interest, in order to develop knowledge and skills in teamwork, problem solving, conflict resolution, communication, and leadership [55]. It would also be important to create a working environment and procedures that reinforce the interdisciplinary approach, which is the primary objective of professionals working together in integrated teams [11]. In addition, nurses need to assume responsibility for activities related to the ‘performance management’ and ‘transparent entrepreneurship’ dimensions. Nurses appear not to be very interested in aspects of functional integration that nevertheless affect organizational factors (economic, political, technical, and legal) and could have an impact on the provision of patient-family centered care.

The study setting presented organizational and professional characteristics similar to those found in all HSSCs in Quebec, which suggests that the results would be transferable to other settings. Studying four different care pathways situated at different phases of development provided an opportunity to validate the measurement instrument while also contributing to a better understanding of the integration process from a nursing perspective. Nevertheless, two key limitations should be kept in mind when interpreting the results of this study. The first is the size of the sample, which was 107 respondents. The small number of staff from each pathway or in different respondent groups (e.g. by training) limited our ability to perform certain analyses that might have added to the results. Still, when using corrected data (n = 200, questionnaires sent) rather than raw data (n = 107, questionnaires completed), applying a hypergeometric distribution at a 95% threshold, with a unilateral left-sided confidence interval providing a true minimal value, the results are the same, with the POS pathway positioned at a more advanced phase, the ASE pathway at an intermediate phase, and the MHS and COPD pathways at a less advanced phase. The second limitation involves the fact that the study was concentrated in a single establishment, such that we cannot make assumptions regarding the generalizability of the results, even if the selected establishment is dealing with the same challenges facing all the others. Moreover, while this study focuses on enriching our understanding of the nursing perspective, a research approach taking into account the perspectives of a wider spectrum of actors (other disciplines and professions, as well as managers) might lead to an even deeper understanding of issues underlying integration. In short, it would be valuable for future studies to apply the measurement instrument to a greater number of nurses, to a range of interdisciplinary team members, including medical staff, professionals, and managers, as well as to a range of establishments and care pathways, to ensure theoretical validation of results, to further develop integration concepts and development mechanisms, to widen the scope of results, and to track the evolution of integration projects within organizations.

## Conclusions

Even with the above-mentioned limitations, this study revealed a gap between the evolution of nursing practice and the introduction of changes aimed at increasing service integration. A significant portion of the activities needed to support service integration are not being implemented in nursing practice. Thus, certain essential dimensions are absent in some pathways or are being adopted at a slower pace than in others, such that some pathways are not very advanced in the integration process. These results suggest that particular efforts should be made to support the development and coordination of care pathways and the renewal of nursing practice in a service integration context. These investments should concentrate on, among other things, support for standard practices geared toward integration; financial and human resources dedicated to new positions or key functions for the development of structural or organizational integration mechanisms; development of a cross-cutting vision of integration shared by the management teams of the various service-programs; and growth of a continuous learning and training culture centering on collaborative approaches, interdisciplinarity, and information-sharing mechanisms.

## Additional file

Additional file 1:
**Data collection instrument - study questionnaire.**

## Abbreviations

ASE: Autonomy support for the elderly (*SoAu: soutien à l’autonomie*)
CCM: Chronic care model
CH: Hospital
CHSLD: Residential and long-term care center
CLSC: Local community services center
COPD: Chronic obstructive pulmonary disease (*Mpoc: maladies pulmonaires obstructives chroniques*)
FMG: Family medicine group
HSSC: Health and social services center
ISN: Integrated services network
LSN: Local services network
MHS: Mental health services (*SaMe: santé mentale*)
MSSS: Ministry of health and social services
IIP: Interdisciplinary intervention plan
POS: Palliative oncology services (*PaOn: soins palliatifs/oncologiques*)
