# Supplementary material for: Implementation of integrated services networks in Quebec and nursing practice transformation: convergence or divergence?
Source: BMC Health Serv Res. 2015 Mar 3;15:84. doi: 10.1186/s12913-015-0720-8 (PMC4359500; doi:10.1186/s12913-015-0720-8)
Supplement: Additional file 1: — Data collection instrument - study questionnaire. [file 12913_2015_720_MOESM1_ESM.docx]

**
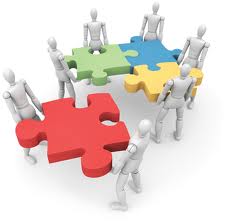
**

**Questionnaire**

***« Ma pratique clinique en contexte d’intégration des soins »^[[1]](#footnote-1)^***

Bonjour. Ce questionnaire comprend trois sections; 1) Des données sociodémographiques nécessaires à l’établissement de statistiques, 2) l’outil de Minkman Ahaus et coll., (2011) permettant de caractériser votre pratique dans un contexte de continuum des soins offert à la clientèle et 3) l’outil inspiré de Vandenberghe et coll., (2004) qui consiste à caractériser votre niveau de bien-être au travail.

**SECTION 1 : QUESTIONNAIRE « MES DONNÉES SOCIODÉMOGRAPHIQUES^[[2]](#footnote-2)^ »**

Veuillez cochez les cases appropriées ou compléter les espaces réservées le cas échéant:

1. Sexe : F: □ M: □

2. Votre titre d’emploi actuel :

| Inf. auxiliaire : □ | Inf. soignante : □ | Inf. clinicienne : □ | Conseillère clinicienne: □ | Inf. pivot : □ |
| --- | --- | --- | --- | --- |
| Inf. de liaison : □ | Inf. gestionnaire : □ | Chargée de projet : □ | Coordonnatrice : □ | Directrice : □ |
| Monitrice clinique : □ | Inf. praticienne : □ | Gestionnaire : □ | Autres : précisez : | |

3. Votre plus haut niveau de formation complétée:

| École d’infirmière : □ | Collégiale : □ | Certificat : □ | Baccalauréat : □ | Maîtrise : □ |
| --- | --- | --- | --- | --- |
| DEC-BAC : □ | Microprogramme : □ | DESS : □ | Doctorat : □ | Autre : précisez : |
| Formation en cours : | | | | |

4. Ancienneté au CSSSSL : nombre d’années _______ nombre de mois _____________

5. Ancienneté dans votre continuum de soins actuel : nombre d’années _______ nombre de mois _____________

6. Année d’obtention de votre permis de pratique : ______________

7. Quart de travail principal: jour □ soir □ nuit □ rotation □

| Hôpital: □ | CLSC : □ | Centre d’hébergement : □ | Centre de jour : □ | GMF : □ |
| --- | --- | --- | --- | --- |
| Clinique externe : □ | Ambulatoire : □ | Centre administratif : □ | Maison de soins palliatifs : □ | |
| Autre : précisez : | | | | |

8. Lieu principal de votre pratique :

9. Direction « programmes et services » d’attache :

| Enfance, jeunesse, famille : □ | Soutien à l’autonomie : □ | Santé mentale adulte : □ | Santé physique (Palliatif/Onco): □ | Services généraux (MPOC, Concerto): □ |
| --- | --- | --- | --- | --- |

10. Qui est votre clientèle cible : ______________________________________

**SECTION 2 : QUESTIONNAIRE « MODÈLE DE DÉVELOPPEMENT DE L’INTÉGRATION DES SOINS (MDIS) »**

**© M. Minkman, 2011, traduit et adapté par C. Longpré, 2011**

Le Modèle de développement de l’intégration des soins (MDIS) ci-dessous permet de décrire les caractéristiques du continuum de soins dans lequel vous travaillez, (**partie a**), décrire les éléments importants de la pratique clinique permettant de développer des soins en continus et uniformisés (mieux intégrés) sur l’ensemble du continuum de soins (**partie b**) et situer le développement de ce continuum de soins (**partie c**).

**Partie a :** Caractéristiques du continuum de soins pouvant être suivi par VOTRE clientèle pour l’ensemble de son épisode de soins; Veuillez cochez **TOUTES** les cases appropriées ou compléter les espaces réservées le cas échéant.

- 1. Quels sont les services ou fournisseurs de soins faisant parties de l’ensemble de ce continuum de soins ?

| Soins palliatifs, domicile □ | Centre d’hébergement □ | Soins à domicile □ | Service scolaire □ | Clinique privée □ |
| --- | --- | --- | --- | --- |
| Soins palliatifs, A. Dion □ | Résidence privée □ | CLSC □ | Organisme communauté □ | Hôpital □  Unité : ______  Unité : ______  Unité : ______ |
| Soins palliatifs, CH □ | Centre de jour □ | Centre de réadaptation □ | Service ambulatoire □ |  |
| GMF, UMF □ | Service santé mentale □ | Clinique externe □ | Maison convalescence □ |  |
| Autres : | | | | |

b. Quels sont les services (ou établissements) avec lesquels vous êtes en relation (ex : communication) dans le cadre de vos fonctions ?

| Soins palliatifs, domicile □ | Centre d’hébergement □ | Soins à domicile □ | Service scolaire □ | Clinique privée □ |
| --- | --- | --- | --- | --- |
| Soins palliatifs, A. Dion □ | Résidence privée □ | CLSC □ | Organisme communauté □ | Hôpital □  Unités : ______  Unités : ______  Unités : ______ |
| Soins palliatifs, CH □ | Centre de jour □ | Centre de réadaptation □ | Service ambulatoire □ |  |
| GMF, UMF □ | Service santé mentale □ | Clinique externe □ | Maison convalescence □ |  |
| Autres : | | | | |

c. Qui sont les professionnels faisant parties de l’ensemble de ce continuum de soins ?

| Omnipraticiens : □ | Inf. soignante : □ | Inf. clinicienne : □ | Conseillère clinicienne: □ | Inf. pivot : □ | Autres : précisez : |
| --- | --- | --- | --- | --- | --- |
| Inf. de liaison : □ | Inf. gestionnaire : □ | Chargée de projet : □ | Inhalothérapeutes : □ | Directrice : □ |  |
| Monitrice clinique: □ | Inf. praticienne : □ | Gestionnaire : □ | Pharmacien : □ | Diététiste : □ |  |
| Orthophoniste : □ | Physiothérapeute : □ | Ergothérapeute : □ | Travailleur social : □ | Psychologue : □ |  |

d. Quels sont les professionnels avec lesquels vous êtes en relation (ex : communication) dans le cadre de vos fonctions ?

| Omnipraticiens : □ | Inf. soignante : □ | Inf. clinicienne : □ | Conseillère clinicienne: □ | Inf. pivot : □ | Autres : précisez : |
| --- | --- | --- | --- | --- | --- |
| Inf. de liaison : □ | Inf. gestionnaire : □ | Chargée de projet : □ | Inhalothérapeutes : □ | Directrice : □ |  |
| Monitrice clinique: □ | Inf. praticienne : □ | Gestionnaire : □ | Pharmacien : □ | Diététiste : □ |  |
| Orthophoniste : □ | Physiothérapeute : □ | Ergothérapeute : □ | Travailleur social : □ | Psychologue : □ |  |

2. À propos de la coordination de ce continuum de soins :

a. Êtes-vous impliquée (ou avez-vous déjà été impliquée) dans un groupe de travail qui se consacre au développement ou au fonctionnement de ce continuum de soins ? : OUI □ NON □

b. Si OUI quelle est (ou qu’elle a été) la nature de votre implication ? ____________________________________________________

**Partie b :** Description de votre pratique

Voici les thèmes et les éléments du MDSI qui caractérisent la pratique en contexte de continuum de soins. Pour chacun des éléments, indiquez d’abord si cet élément est PERTINENT (cochez OUI ou NON) pour votre service (VOTRE MILIEU DE TRAVAIL) faisant partie du continuum de soins et indiquez ensuite, si l’élément est PRÉSENT, soit en vigueur et pratiqué dans votre service (cochez OUI ou NON). Suivant chaque thème se trouve une question pour laquelle vous devez indiquez votre niveau d’accord (pas du tout d’accord à tout à fait d’accord).

**Thème 1 : Soins centrés sur le client/famille (C/F) :** Ce thème réfère à l’échange d’information concernant les soins du C/F entre les partenaires de soins^[[3]](#footnote-3)^ et le C/F lui-même, sur l’ensemble du continuum de soins (porte d’entrée, soins, transferts, soutien à la prise en charge, etc.). Réfère aussi à la prestation de soins individualisés et adaptés aux besoins du C/F.

| **Description des éléments de la pratique** | **Pertinent pour mon service** | | **Présent dans mon service** | |
| --- | --- | --- | --- | --- |
|  | OUI | NON | OUI | NON |
| a. Transmettre, en collaboration avec les partenaires de soins, de l’information au C/F qui soit centrée sur sa situation |  |  |  |  |
| b. Partager entre les partenaires de soins de l’information sur le C/F |  |  |  |  |
| c. Déterminer avec les partenaires de soins, du plan de soins requis (PTI et PII) pour le C/F (cas complexe) |  |  |  |  |
| d. Utiliser des moyens pour favoriser l’auto-prise en charge du C/F au sein du continuum de soins |  |  |  |  |
| e. Instaurer des outils de communication ou système d’information clinique ex : PTI, PII, informatique, qui soutiennent le processus de soins tout au long du continuum de soins |  |  |  |  |
| f. Ajuster les soins tout au long du continuum aux besoins particuliers du C/F |  |  |  |  |
| g. Offrir au C/F un point d’accès (guichet) à l’information sur ses soins |  |  |  |  |
| h. Utiliser un protocole clinique permettant d’assurer le suivi systématique du C/F |  |  |  |  |
| i. Utiliser des protocoles soins standardisés (ex; suivi systématique) adaptés à des groupes de C/F ayant des besoins précis |  |  |  |  |
| Thème 1 :  En lien avec votre milieu de pratique; le développement de ce continuum de soins a contribué, contribue ou contribuera au développement de ces éléments de la pratique (du thème 1) ? | Pas du tout d’accord □  Désaccord □  Ni l’un ni l’autre □  D’accord □  Tout à fait d’accord □ | | | |

**Thème 2: Système de prestation des soins:** Ce thème vise la logistique du continuum de soins pour le C/F : les mécanismes de coordination et les procédures visant à optimiser l’ensemble des soins, l’atteinte de consensus et d’ententes (p. ex., sur la logistique, partage de l’expertise, échange d’information, outils de soins) nécessaires de l’admission du C/F jusqu’à la fin de son épisode de soins.

| **Description des éléments de la pratique** | **Pertinent pour mon service** | | **Présent dans**  **mon service** | |
| --- | --- | --- | --- | --- |
|  | OUI | NON | OUI | NON |
| a. S’entendre quant à la référence et au transfert du C/F au travers du continuum de soins (ex : d’un milieu à l’autre) |  |  |  |  |
| b. Identifier des moyens pour échanger de l’information sur le C/F au travers du continuum de soins (ex; PTI) |  |  |  |  |
| c. Utiliser un dossier commun pour le C/F, qui soit accessible à tous les partenaires de soins sur tout le continuum de soins |  |  |  |  |
| d. S’entendre et respecter les modalités d’échange d’information sur le C/F |  |  |  |  |
| e. Accéder aux bases de données de tous les partenaires de soins du continuum de soins |  |  |  |  |
| f. Offrir un service de « gestion de cas » pour le C/F dont les besoins sont complexes |  |  |  |  |
| g. S’entendre sur les aspects logistiques du continuum de soins (p. ex., temps d’attente et délais de tx.) |  |  |  |  |
| h. Utiliser des plans de soins et de traitements communs sur tout le continuum de soins |  |  |  |  |
| i. Utiliser un système uniforme d’identification du C/F au sein du continuum de soins |  |  |  |  |
| j. S’entendre entre partenaires de soins sur les consultations d’experts requis pour le C/F |  |  |  |  |
| k. S’entendre entre partenaires de soins sur la gestion et le respect des soins centrés sur les préférences du C/F |  |  |  |  |
| l. S’entendre entre partenaires de soins sur le calendrier des examens et des traitements du C/F |  |  |  |  |
| m. S’entendre entre partenaires de soins sur la planification du congé |  |  |  |  |
| n. Élaborer des critères d’inclusion et de cheminement du C/F dans le continuum de soins |  |  |  |  |
| o. S’entendre entre partenaires de soins sur la prestation de soins aux C/F inscrits sur une liste d’attente |  |  |  |  |
| p. Recourir à une ou des infirmières spécialisées en regard des soins à offrir au sein du continuum de soins |  |  |  |  |
| q. S’entendre entre partenaires de soins quant à la référence du C/F avec des ressources communautaires |  |  |  |  |
| r. Élaborer des critères d’évaluation uniformes permettant d’évaluer l’urgence de la situation de santé du C/F |  |  |  |  |
| Thème 2 :  En lien avec votre milieu de pratique, le développement de ce continuum de soins a contribué, contribue ou contribuera au développement de ces éléments de la pratique (thème 2) ? | Pas du tout d’accord □  Désaccord □  Ni l’un ni l’autre □  D’accord □  Tout à fait d’accord □ | | | |

**Thème 3 : Gestion du rendement :** Ce thème représente la mesure et l’analyse des résultats des soins prodigués au sein du continuum de soins, à partir des objectifs de rendement fixés, l’utilisation d’indicateurs uniformisés, les rapports accidents/incidents et la rétroaction (partager les résultats suite à l’évaluation). Il tient compte de l’évaluation faite par le C/F, des résultats organisationnels et du rendement financier.

| **Description des éléments de la pratique** | **Pertinent pour mon service** | | **Présent dans**  **mon service** | |
| --- | --- | --- | --- | --- |
|  | OUI | NON | OUI | NON |
| a. Définir des indicateurs de rendement^[[4]](#footnote-4)^ des soins offerts en continuum |  |  |  |  |
| b. Offrir aux partenaires de soins d’où vient le C/F (avant son transfert sur mon unité), de l’information sur son évolution |  |  |  |  |
| c. Recueillir, au sein du continuum, des données sur les indicateurs de rendement des soins (état de santé, qualité de vie) |  |  |  |  |
| d. Recueillir, au sein du continuum, des données sur les logistiques du continuum (ex., achalandage, attente, délais Tx) |  |  |  |  |
| e. Utiliser les commentaires des partenaires de soins au sein du continuum pour améliorer les soins |  |  |  |  |
| f. S’entendre sur l’utilisation uniforme des indicateurs de rendement au sein du continuum de soins |  |  |  |  |
| g. Faire le suivi des résultats obtenus au cours du développement du continuum de soins |  |  |  |  |
| h. Établir des objectifs de qualité des soins pour l’ensemble du continuum de soins |  |  |  |  |
| i. Assurer le suivi des rapports d’accident/incident en lien avec le continuum (ex : transferts inopportuns, ↓ information) |  |  |  |  |
| j. Suivre une méthode systématique d’évaluation des approches utilisées (ex : prestation de soins) et des résultats obtenus |  |  |  |  |
| k. Faire le suivi des évaluations faites par le client et des données de satisfaction de la clientèle |  |  |  |  |
| l. Recueillir, au sein du continuum, des données sur le rendement financier du continuum de soins |  |  |  |  |
| m. Démontrer aux partenaires de soins les effets du continuum sur les soins qui sont offerts |  |  |  |  |
| n. Exercer un suivi visant à déterminer si les soins prodigués sont conformes aux résultats probants |  |  |  |  |
| o. Établir des objectifs de qualité des soins offerts par les partenaires de soins |  |  |  |  |
| p. Déployer des équipes chargées de l’amélioration du processus de soins au travers du continuum de soins |  |  |  |  |
| Thème 3 :  En lien avec votre milieu de pratique, le développement de ce continuum de soins a contribué, contribue ou contribuera au développement de ces éléments de la pratique (thème 3) ? | Pas du tout d’accord □  Désaccord □  Ni l’un ni l’autre □  D’accord □  Tout à fait d’accord □ | | | |

**Thème 4 : Qualité des soins :** Ce thème est centré sur le développement d’un continuum de soins interdisciplinaires conformément à des normes et à des recommandations fondées sur des résultats probants et en fonction des besoins et des préférences du C/F.

| **Description des éléments de la pratique** | **Pertinent pour mon service** | | **Présent dans**  **mon service** | |
| --- | --- | --- | --- | --- |
|  | OUI | NON | OUI | NON |
| a. Évaluer systématiquement les besoins du client au travers du continuum de soins |  |  |  |  |
| b. Développer le travail en équipe interdisciplinaire au sein du continuum de soins |  |  |  |  |
| c. Assurer la participation de représentants de la clientèle aux projets d’amélioration du continuum de soins |  |  |  |  |
| d. Se conformer aux recommandations et aux normes de pratique fondées sur des résultats probants |  |  |  |  |
| e. Assurer la participation de représentants de clientèle aux évaluations du rendement du continuum de soins |  |  |  |  |
| Thème 4 :  En lien avec votre milieu de pratique, le développement de ce continuum de soins a contribué, contribue ou contribuera au développement de ces éléments de la pratique (thème 4) ? | Pas du tout d’accord □  Désaccord □  Ni l’un ni l’autre □  D’accord □  Tout à fait d’accord □ | | | |

**Thème 5 : Apprentissage axé sur les résultats :** Ce thème réfère à l’établissement d’une culture d’amélioration continue des résultats obtenus dans le continuum de soins. Essentiels à l’amélioration continue: définition des objectifs de collaboration, identification des points à améliorer dans les soins et de stratégies d’apprentissage, échange de connaissances, mesures incitatives pour encourager le rendement amélioré.

| **Description des éléments de la pratique** | **Pertinent pour mon service** | | **Présent dans**  **mon service** | |
| --- | --- | --- | --- | --- |
|  | OUI | NON | OUI | NON |
| a. Promouvoir une culture d’amélioration continue dans le continuum de soins |  |  |  |  |
| b. Évaluer les soins prodigués en collaboration avec les partenaires de soins |  |  |  |  |
| c. Partager les bienfaits de la collaboration pour chacun des partenaires de soins |  |  |  |  |
| d. Évaluer en collaboration avec les partenaires de soins, les points à améliorer dans les soins |  |  |  |  |
| e. Partager des connaissances entre partenaires sur l’organisation efficace des services dans le continuum de soins |  |  |  |  |
| f. Favoriser les discussions portant sur les améliorations pouvant être apportées par les partenaires de soins |  |  |  |  |
| g. Échanger de l’information entre partenaires relativement au processus de soins |  |  |  |  |
| h. Offrir des mesures incitatives aux partenaires de soins qui encouragent à atteindre des objectifs de qualité de soins |  |  |  |  |
| i. Utiliser les connaissances et l’information disponibles (indicateurs) pour diriger et coordonner le continuum de soins |  |  |  |  |
| j. Accéder à des programmes de formation et à des occasions d’apprentissage pour les partenaires de soins |  |  |  |  |
| k. Diffuser aux partenaires de soins, les résultats de la réalisation des objectifs du continuum |  |  |  |  |
| l. Promouvoir les échanges entre les partenaires en vue d’apporter des innovations aux soins offerts dans le continuum |  |  |  |  |
| Thème 5 :  En lien avec votre milieu de pratique, le développement de ce continuum de soins a contribué, contribue ou contribuera au développement de ces éléments de la pratique (thème 5) ? | Pas du tout d’accord □  Désaccord □  Ni l’un ni l’autre □  D’accord □  Tout à fait d’accord □ | | | |

**Thème 6 : Travail en équipe interdisciplinaire :** Ce thème représente le travail en interdisciplinarité, auprès d’un groupe de C/F défini, qui constitue la cible de travail des professionnels appelés à collaborer au sein de ces équipes organisées et intégrées au continuum de soins.

| **Description des éléments de la pratique** | **Pertinent pour mon service** | | **Présent dans**  **mon**  **service** | |
| --- | --- | --- | --- | --- |
|  | OUI | NON | OUI | NON |
| a. Définir avec les partenaires de soins, le groupe de C/F ciblé par le continuum de soins |  |  |  |  |
| b. Travailler en équipes interdisciplinaire |  |  |  |  |
| c. S’entendre sur la disponibilité et l’accessibilité des partenaires de soins |  |  |  |  |
| Thème 6 :  En lien avec votre milieu de pratique, le développement de ce continuum de soins a contribué, contribue ou contribuera au développement de ces éléments de la pratique (thème 6) ? | Pas du tout d’accord □  Désaccord □  Ni l’un ni l’autre □  D’accord □  Tout à fait d’accord □ | | | |

**Thème 7 : Rôles et responsabilités :** Ce thème reflète la nécessité de clarifier les rôles et les responsabilités de chacun des partenaires dans le continuum de soins. Les visées sont la collaboration efficace et l’attribution appropriée des tâches de coordination.

| **Description des éléments de la pratique** | **Pertinent pour mon service** | | **Présent dans**  **mon service** | |
| --- | --- | --- | --- | --- |
|  | OUI | NON | OUI | NON |
| a. Échanger entre partenaires de soins sur les rôles et les responsabilités de chacun |  |  |  |  |
| b. Effectuer des ajustements au besoin en lien avec les rôles, entre partenaires de soins |  |  |  |  |
| c. S’assurer que les partenaires de soins connaissent les rôles et responsabilités de chacun |  |  |  |  |
| d. Assigner un coordonnateur de soins à l’ensemble du continuum de soins |  |  |  |  |
| e. Définir les rôles et les responsabilités des membres de l’équipe interdisciplinaire |  |  |  |  |
| f. Favoriser des rencontres entre les partenaires sur l’ensemble du continuum de soins |  |  |  |  |
| g. S’entendre sur la manière de présenter et d’intégrer de nouveaux partenaires dans le continuum de soins |  |  |  |  |
| h. Diriger le continuum de soins par des personnes exerçant des fonctions de coordination |  |  |  |  |
| Thème 7 :  En lien avec votre milieu de pratique, le développement de ce continuum de soins a contribué, contribue ou contribuera au développement de ces éléments de la pratique (thème 7) ? | Pas du tout d’accord □  Désaccord □  Ni l’un ni l’autre □  D’accord □  Tout à fait d’accord □ | | | |

**Thème 8 : Engagement :** Ce thème est axé sur l’engagement en regard à des objectifs définis par le continuum de soins, l’engagement à la collaboration et la volonté d’y participer et la connaissance quant à la nature du travail dans un continuum de soins.

| **Description des éléments de la pratique** | **Pertinent pour mon service** | | **Présent dans**  **mon service** | |
| --- | --- | --- | --- | --- |
|  | OUI | NON | OUI | NON |
| a. Définir les objectifs de collaboration dans le continuum de soins |  |  |  |  |
| b. Signer des ententes de collaboration entre les partenaires de soins |  |  |  |  |
| c. Assurer l’engagement du leadership des partenaires de soins impliqués dans le continuum de soins |  |  |  |  |
| d. Décrire les rôles et responsabilités des leaders, coordonnateurs et comités consultatifs au sein du continuum |  |  |  |  |
| e. Formaliser les liens d’interdépendance entre partenaires de soins et établissements de soins (continuum) |  |  |  |  |
| f. Renforcer l’engagement au principe de collaboration, orienté vers le continuum de soins |  |  |  |  |
| g. Tenir des rencontres entre les dirigeants des organisations faisant partie du continuum de soins |  |  |  |  |
| h. Conclure des ententes quant aux domaines de soins respectifs entre partenaires de soins (qui fait quoi) |  |  |  |  |
| i. Promouvoir la confiance entre partenaires de soins |  |  |  |  |
| j. Sensibiliser les partenaires de soins à la nature du travail dans un continuum de soins |  |  |  |  |
| k. Rencontrer des intervenants externes : agences gouvernementales, organismes communautaires, etc. |  |  |  |  |
| Thème 8 :  En lien avec votre milieu de pratique, le développement de ce continuum de soins a contribué, contribue ou contribuera au développement de ces éléments de la pratique (thème 8) ? | Pas du tout d’accord □  Désaccord □  Ni l’un ni l’autre □  D’accord □  Tout à fait d’accord □ | | | |

**Thème 9 : Entreprenariat :** Ce thème fait place à l’innovation (expérimentation), les responsabilités de leadership en matière de rendement et les accords financiers couvrant le continuum de soins.

| **Description des éléments de la pratique** | **Pertinent pour mon service** | | **Présent dans**  **mon service** | |
| --- | --- | --- | --- | --- |
|  | OUI | NON | OUI | NON |
| a. S’engager mutuellement à l’atteinte des objectifs cliniques visés par le fonctionnement en continuum de soins |  |  |  |  |
| b. Utiliser un langage commun à l’intérieur du continuum de soins |  |  |  |  |
| c. S’entendre sur l’établissement d’un budget financier pour le continuum de soins |  |  |  |  |
| d. Allouer des budgets financiers pour la mise en œuvre et le maintien du continuum de soins |  |  |  |  |
| e. Faire participer les dirigeants aux efforts d’amélioration dans le continuum de soins |  |  |  |  |
| f. Promouvoir un environnement ouvert qui encourage l’expérimentation et les projets pilotes et la recherche |  |  |  |  |
| g. Disposer d’un financement global à répartir sur l’ensemble du continuum de soins |  |  |  |  |
| Thème 9 :  En lien avec votre milieu de pratique, le développement de ce continuum de soins a contribué, contribue ou contribuera au développement de ces éléments de la pratique (thème 9) ? | Pas du tout d’accord □  Désaccord □  Ni l’un ni l’autre □  D’accord □  Tout à fait d’accord □ | | | |

**Selon vous, y aurait-il d’autres éléments qui contribuent à une meilleure pratique en continuum de soins? (QUESTION OUVERTE)**

____________________________________________________________________________________________________________________________________________________________________________________________________________________________________________________________________________________________________________________________________________________________

Commentaires :

Merci de votre collaboration

_________________________________

Caroline Longpré (cand. Ph.D.) [caroline.longpre@umontreal.ca](mailto:caroline.longpre@umontreal.ca)

1. Dans le cadre de l’étude : « *La pratique clinique et le bien-être au travail des infirmières en contexte d’intégration des soins* »

   Caroline Longpré, *cand. Ph.D.,* Sc. Inf., option : Administration des services infirmiers, Université de Montréal [↑](#footnote-ref-1)
2. Le féminin est employé tout au long du questionnaire dans le seul but d’alléger le texte [↑](#footnote-ref-2)
3. Le terme « partenaires de soins » inclus les soins infirmiers, médicaux et professionnels impliqués pour le C/F sur l’ensemble du continuum de soins [↑](#footnote-ref-3)
4. Des indicateurs de rendement ou de performance de l’organisation tels que la qualité des soins, l’offre de service, le financement, etc.) [↑](#footnote-ref-4)
